# Contexts as Shared Commitments

**DOI:** 10.3389/fpsyg.2015.01932

**Published:** 2015-12-22

**Authors:** Manuel García-Carpintero

**Affiliations:** LOGOS-Departament de Lògica, Història i Filosofia de la Ciència, Universitat de BarcelonaBarcelona, Spain

**Keywords:** context, presupposition, accommodation, meaning normativity, rules

## Abstract

Contemporary semantics assumes two influential notions of context: one coming from Kaplan (1989), on which contexts are sets of predetermined parameters, and another originating in Stalnaker (1978), on which contexts are sets of propositions that are “common ground.” The latter is deservedly more popular, given its flexibility in accounting for context-dependent aspects of language beyond manifest indexicals, such as epistemic modals, predicates of taste, and so on and so forth; in fact, properly dealing with demonstratives (perhaps ultimately all indexicals) requires that further flexibility. Even if we acknowledge Lewis (1980)'s point that, in a sense, Kaplanian contexts already include common ground contexts, it is better to be clear and explicit about what contexts constitutively are. Now, Stalnaker (1978, 2002, 2014) defines context-as-common-ground as a set of propositions, but recent work shows that this is not an accurate conception. The paper explains why, and provides an alternative. The main reason is that several phenomena (presuppositional treatments of pejoratives and predicates of taste, forces other than assertion) require that the common ground includes non-doxastic attitudes such as appraisals, emotions, etc. Hence the common ground should not be taken to include merely contents (propositions), but those together with attitudes concerning them: *shared commitments*, as I will defend.

## Two notions of context

As Stalnaker (2014, pp.13–34) reminds us, in formal semantics/pragmatics there have been two prominent theoretical articulations of the intuitive notion of a context—a concrete situation relative to which linguistic exchanges take place. The first is the one described in Kaplan's (1989) work, by means of which Kaplan's important notion of *character* (the linguistic meaning of context-dependent expressions) is defined. On this view, a context is a sequence of items on which the content of a sentence (“what is said” with it) might depend, given the character of some of the expressions in it. Thus, a context includes a speaker, the value of the character of “I”; a time, the value of the character of “now”; a place, the value of the character of “here”; a possible world, the value of the character of “actual.” In contemporary intensional semantics, this is modeled as a *centered possible world*—a possible world together with a designated time and subject.

The second is the notion characterized in Stalnaker's (1978) influential work on presupposition and assertion: “a body of information that is available, or presumed to be available, as a resource for communication” (Stalnaker, 2014, p. 24). This is modeled as the “context set”—the set of possible worlds compatible with the presumed common knowledge of the participants^1^. This second notion is supposed to encompass the previous one, because the information needed to interpret indexicals (who the speaker is, what the time is, the place and the world in which the exchange takes place) is included in the context set. This raises delicate issues, not unrelated to the ones that I will be discussing, concerning how the “propositions” that make up contexts-as-common-ground should be understood for this claim to be justified; but it seems intuitively acceptable as a starting point^2^.

At first sight, the second conception is considerably more flexible than the first one, and as a result more adequate as a theoretical tool. In addition to “pure indexicals” like those already mentioned, there are demonstratives such as “he,” “you,” “that”; and their contribution to content appears to depend not on “objective” features of the concrete situation, but on what the participants take for granted (about who is the salient/demonstrated male or female, etc.) when they are uttered. To some researchers, including the present author, “answering machines” and related examples suggest that the divide between pure indexicals and demonstratives is spurious (cf. Cohen and Michaelson, 2013 and references there, although the authors do not subscribe to those views). And most linguists also contend that the distinction between *deictic* uses of indexicals, whose reference is determined by means of demonstrations, and *anaphoric* uses, determined rather by means of their links to the previous discourse, does not draw a genuine semantic boundary. As Heim and Kratzer (1998, p. 240) put it, “anaphoric and deictic uses seem to be special cases of the same phenomenon: the pronoun refers to an individual which, for whatever reason, is highly salient at the moment when the pronoun is processed.” To the extent that we are clear as to how the information that *o* is the speaker comes to be in the context-as-common-ground so that utterances of “I” can be interpreted, it does not seem more problematic to understand how the information that *o* is the demonstrated male comes to be there.

Lewis (1980, pp. 85–86) points out, however, that the Kaplanian notion of context can also be sensibly taken to encompass the Stalnakerian one, and the previous points with it:

That is not to say that the only features of context are time, place, and world. There are countless other features, but they do not vary independently. They are given by the intrinsic and relational character of the time, place, and world in question. The speaker of the context is the one who is speaking at that time, at that place, at that world …The audience, the standards of precision, the salience relations, the presuppositions…of the context are given less directly. They are determined, so far as they are determined at all, by such things as the previous course of the conversation that is still going on at the context, the states of mind of the participants, and the conspicuous aspects of their surroundings.

Thus, the two notions of context might be perfectly compatible, “complementary, rather than alternative theories of the same thing” (Stalnaker, 2014, p. 16). For present purposes, however, I'll assume the Stalnakerian one; even if Lewis is right, it has at least the advantage of allowing for a more perspicuous presentation of the relevant features on which our theoretical proposals rely^3^.

As we have seen, the Stalnakerian notion is characterized as a set of propositions, or contents. The point I want to make in this article is that we should think of them as having instead a richer structure—more specifically, as having illocutionary features, understood in non-psychological, normative terms^4^. I will argue that it should not be understood as a set of propositions (or other representational contents) that are (presumed to be) mutually known, or mutually believed, but, more generally, as a class of shared propositional commitments—some in the belief-mode, but some in other illocutionary modes too^5^.

The argument in the following pages proceeds by laying down five illustrative examples, observing that each of them constitutes a particular instance of the main claim just stated. They are: the contribution to the determination of *what is said* of a “question under assumption”; the interpretation of directives; the interpretation of pejoratives and slurs; the semantic of predicates of taste; the interpretation of fictions. Before going into the discussion of the examples, however, I need to say something about meaning and norms—both discourse norms, such as conversational norms and rules of accommodation, and illocutionary norms.

I should admit at the outset that the point I want to make should not be controversial, and in fact it is in a way obvious to researchers in this field. It is enough to pay attention to the fact that questions and commands make contributions of their own to the context in order to realize this. It is sometimes noted, and just put aside for reasons of expediency, because the semantics of declaratives is more familiar and well-studied. However, I will show that not having it clearly in mind leads to faulty arguments and overlooked possibilities. Section Example 3: Pejoratives and Slurs below on pejoratives is thus the core of the paper. In defending his truth-conditional account of pejoratives that I will question there, Hom (2012) approvingly quotes MacFarlane (2011):

The beauty of truth-conditional semantics is that it provides a common currency that can be used to explain indefinitely many interaction effects in a simple and economical account. We should be prepared to accept a messy, non-truth-conditional account …only if there is no truth-conditional account that explains the data.

The uncontroversial point that my two initial examples make shows this rhetoric to be highly problematic; in addition, these two initial examples will show how general the point is, in fact affecting all ordinary discourses. The three final examples show that we ignore it at our peril, starting with the very case for which Hom invokes the rhetoric of the methodological priority of the familiar semantics for declaratives.

## Meaning and norms

In the previous section, I contrasted two different ways of thinking of contexts, and favored the Stalnakerian one. In this section I will discuss another contrast, between normative and descriptive, non-normative views of meaning, and I will indicate why I favor the former.

In recent work already mentioned, Stalnaker (2014, pp. 36–37) contrasts two more different ways of thinking of contexts, which, as he points out, reflect the contrasting ways in which Austin and Grice thought of speech acts. Austin (1962) suggests thinking of them as social practices constituted by social norms, usually established and maintained by conventions; Grice (1957) takes them instead to be definable in natural, psychological terms, appealing to a peculiar kind of reflexive intention. Stalnaker's own views favor the latter sort of account; Lewis (1979) offers a model well adapted to the former. With respect to this issue, I depart from Stalnaker's views and favor the ones he rejects.

What is at stake in such debates? For present purposes, I'll just mention two relevant concerns that Austinians have with the Gricean account, which I take very seriously. On the negative side, Austinians emphasize that speech acts might well take place even when their authors lack the complex intentions that Griceans posit (Alston, 2000, pp. 48–49). A clerk in an information booth makes an assertion when she utters “the plane will arrive on time,” even though she does not care at all what psychological impact this has on her audience. On the positive side, Austinians emphasize that speech acts are governed by norms, not just “regulative” ones (be clear!, polite!, witty!) but *constitutive* ones, and that this has a stronger impact on the determination of the speech act made than whatever communicative intentions the author had. Thus, for instance, the clerk in the above example might be criticized if she cannot have known the information she provided—we had been reliably told that the plane had only just taken off from the departing airport, and so we reply, “you cannot know that!.”

Williamson (1996/2000) has defended an account of assertion along Austinian lines, on which the following norm (the *knowledge rule*) is constitutive of the act, and individuates it:

(KR) One must [(assert *p*) only if one knows *p*].

Other writers have accepted Williamson's view that assertion is defined by constitutive rules such as KR, but have proposed alternative norms; thus, Weiner (2005) proposes a *truth* rule, TR, and Lackey (2007) a *reasonableness* rule, RBR:

(TR) One must [(assert *p*) only if *p*].
(RBR) One must [(assert *p*) only if it is reasonable for one to believe *p*].

Norms like these are *sui generis*: they do not have their sources in moral or prudential codes, but in specifically illocutionary ones. They are defeasible and *pro tanto*: they can be overridden by stronger norms.

And it is possible to violate them, thereby rendering the acts wrong but occurring: what is constitutive of asserting *p* is not that one knows *p*, but that in performing it one is thereby subject to the requirement that one knows *p*. There are plenty of situations in which *p* is asserted when *p* is false, or the speaker lacks justification for it. The assertion is then wrong, and wrong relative to norms defining the nature of such a speech act.

Stalnaker (1978) provides an account of assertion, and of what I take to be an ancillary speech act, presupposition, in a Gricean spirit, on which a presupposition is a requirement on the context, and an assertion is a proposal to change it by adding to it its content, which will take effect if the assertion is not rejected. He (*ibid*., 87) puts forward several reasons why his suggestion cannot be taken as a definition *sensu stricto*: it is not individuative, in that acts other than assertion are such proposals, and presumably would be circular if taken in that way because it helps itself to the notion of another speech act, *rejection*. He nonetheless shows the account to be able to provide explanations for different phenomena.

One of those is *presupposition accommodation*, as when we decline an invitation by uttering “I cannot come, I have to pick up my wife at the airport.” This will not be felt to be in any way incorrect even in contexts in which it is not mutually known, previous to the utterance, that the speaker is married. Stalnaker's suggestion to account for this relies on the correct point that whether or not the presuppositions of an utterance are satisfied should be checked right after the utterance has been produced. This is so because in many cases it is the very occurrence of the utterance that makes it the case that the context includes the information that must be in it for some presuppositions to be correct. Thus, an utterance *u* of “I am hungry” asserts that *x* is hungry, for some assignment to *x*, and presupposes that *x* is the speaker of *u*; but this latter information comes to be in place concurrently with the utterance. Something similar obtains, according to Stalnaker, in the “my wife” case.

Now, in previous work (García-Carpintero, 2015) I have argued that, although this is correct as far as it goes—so that in standard cases of informative presuppositions they have become common knowledge at “presupposition evaluation time,” so that the common knowledge norm for presuppositions is ultimately not violated—in order to sustain it two assumptions that Stalnaker rejects are needed:^6^ first, that some presuppositions (such as those assumed here for “I” and “the”) are lexically triggered, and an adequate semantics for natural languages should countenance them. Second, that we think of presupposing as an ancillary speech act, understood along normative-Austinian lines—its constitutive norm being that the presupposed content is commonly known.

If those points are right, the model that Lewis (1979) provides for presupposing, asserting, and their interrelated effect on contexts such as accommodation is more appropriate than the psychological one that Stalnaker assumes. As is usual when it comes to understanding normative notions, Lewis takes games as a model, and offers different rules of accommodation for different expressions, understood in normative terms. This model can also be helpfully used in order to properly understand indirect speech acts. Grice (1975) offered a deservedly influential analysis for a very specific case, conversational implicatures, in which assertions are indirectly conveyed by other assertions. The specific maxims that Grice provided were attuned to that case and cannot be generalized. For instance, the maxim of quality (“Try to make your contribution one that is true”) cannot be applied to explain how assertions are indirectly conveyed by questions, because questions are not constitutively either true or false. The Cooperative Principle (“make your conversational contribution such as is required, at the stage at which it occurs, by the accepted purpose or direction of the talk exchange”), from which Grice derives the specific maxims, is a regulative norm that would be involved in any general account of indirect speech acts.

In sum, the view that I will assume henceforth as a point of departure, which my arguments suggest we should improve, has it that contexts are Stalnakerian sets of propositions as opposed to Kaplanian sets of parameters, and that their dynamics is to be understood along Lewisian normative lines, as opposed to Stalnakerian intentional ones.

## Example 1: underdeterminacy and the question under discussion

I will now start developing my argument. In the next two sections I will focus on two cases for which there is widespread agreement that the propositional account of context is inadequate—questions and directives, the former in this and the latter in the next. I will argue that, even though the point that questions need be treated as adding to contexts more than propositions is sufficiently well established already, it has more pervasive consequences than usually acknowledged or even realized.

Some writers (e.g., Alston, 2000, pp. 116–120; Jary, 2010, pp. 15–16; Pagin, 2011, p. 123) defend accounts of assertion that imply that this act cannot be indirectly made, by requiring that an assertion consists of the communication of the proposition *p by means of a sentence that means p*. I think that this incorrectly makes it impossible by definition to make assertions of *p* with sentences that mean something else (or even by fully non-linguistic means): in rhetorically asking “Who the heck wants to read this book?,” I think I am asserting that (to put it mildly) nobody wants to read it. Aside from direct counterexamples like this, we might ask: why would assertion be special, in being the only speech act that cannot be made indirectly? Unless the point generalizes, and no speech act can be made indirectly; but it seems clear that, say, a literal expression of thanks such as “thanks for not browsing our journals” in a newsstand indirectly conveys a request.

Recently, however, other writers have provided arguments that would answer this worry, and hence would support views of assertion along the lines indicated. While Camp (2006) and Lepore and Stone (2010) have discussed the argument for specific cases such as metaphorical assertions, Fricker (2012) advances more general considerations. A point on which these authors rely is that indirectly conveyed claims are too ambiguous or underdetermined in their contents for the speaker to fully commit to them in the way constitutive of assertions^7^.

Notoriously, similar points have been made by so-called “minimalists” about semantic content such as Cappelen and Lepore (2005) and Borg (2012) against so-called “moderate contextualists” such as Bach (1994). Minimalists defend that semantic contents are truth-conditional (i.e., given a specification of a possible world, they deliver a truth-value), but nonetheless context-invariantly determined except when it comes to the value of “pure indexicals” (those for which Kaplanian contexts reserve parameters, “I,” “now,” “today,” and a few more). On the basis of much-debated examples such as “I am ready,” “I am tall,” “I have had breakfast,” or “there is milk in the fridge,” moderate contextualists plausibly contend that compositionally determined semantic contents are truth-conditionally incomplete: they do not yield a truth-value given a possible world. However, context might help to complete them, fixing a fully determinate content that the utterance literally and directly conveys. Against this, minimalists produce slippery slope arguments (Cappelen and Lepore, 2005, pp. 43–44; Borg, 2012, p. 83), allegedly showing that moderate contextualism is an unstable standpoint, ultimately committing their proponents to the much more radical view put forward, say, by Travis (1985), which does away with any recognizable notion of semantic content. These arguments purport to show that moderate contextualists' strategies would leave literal, directly expressed truth-conditional contents wildly underdertermined.

Thus, if these combined appeals to considerations of semantic underdetermination were valid, we would end up with the absurd consequence that the only contents we can ever assert are trivially true claims such as (on Borg's view, in the case of “I am ready”) *that I am ready for something or other* or their trivially false negations. Fortunately, there is a compelling reply, which Schoubye and Stokke (2015) develop in detail for the case of minimalists' criticisms of moderate contextualism. They appeal to Roberts's (2012) proposal, elaborating on previous work by Carlson (1982) and others, that contexts are structured by a “question under discussion” (QUD) for which discussants try to provide adequate answers^8^. The QUD might have been explicitly asked, but it can also be merely implicit; in some cases, it may be very general, including the “Big Question,” *what is the way things are?* Schoubye and Stokke convincingly argue that, taking this into consideration, in many ordinary contexts moderate contextualists are able to provide sufficiently well-determined (i.e., leaving aside the vagueness and lack of specificity in fact existing in ordinary communication) contents literally and directly expressed, completions of the typically non-truth-conditional, compositionally determined meaning of the uttered sentence.

Bergmann (1982) had in fact already made a similar point in defense of metaphorical assertions, which also gives a compelling reply to Fricker (2012) and Lepore and Stone (2010). As she puts it (*ibid*., 231): “without knowing the context in which a metaphor occurs and who its author is, it is impossible to state conclusively what the metaphor “means” without drawing out all that it could mean …But bring in a well-defined context and a real author, and matters may change drastically.” She (*ibid*.) illustrates this with the following example:

Suppose I say to you, after hearing the latest report on Three Mile Island, “As far as I'm concerned, nuclear reactors are time bombs.” You correctly interpret my remark as an assertion to the effect that nuclear reactors are likely to fail, at any moment-of course, with disastrous consequences. A while later you say, “That was an interesting metaphor: nuclear reactors being time bombs. Although I don't think that the guys responsible for those things want people to get killed by them, still it seems that, like people who use time bombs, they have a frightening disregard for human lives.” This, then, is something else that I could have used the metaphor to assert. But it does not follow, from the possibility of using a metaphor to make different assertions, that anyone who does use that metaphor is making all of those assertions.

Bergmann's point can be articulated by means of Schoubye and Stokke's strategy, by taking the specific feature of context required to develop her argument to be a particular QUD. Thus, we can take the QUD implicitly assumed in her example to be something like this: Which consequences should we derive from the Three Mile Island (28/3/1979) accident?

Using current formal semantics frameworks to model questions, Roberts (2012) provides a particular theoretical representation of QUD, which Schoubye and Stokke invoke to develop their point in a sufficiently precise way. I will not go into those details here. It is however clear how these to my mind very plausible views help illustrating the main claim I want to make here. QUDs interact with Stalnakerian contexts in the sort of rule-governed way Lewis (1979) set out to model with his scorekeeping analogy; but they are not such contexts, for they are not propositions. So, to adopt proposals of the sort just outlined requires us to abandon the simple-minded way of thinking of context presented at the outset. We should think of them as more complex, structured into at least two different components endowed with illocutionary features: a class of propositions that should be mutually known, and a class of questions (also commonly known as such in felicitous cases) for which discussants aim in a coordinated way to provide answers^9^.

## Example 2: directives

As I said above, it is relatively uncontroversial that, while questions make contributions to context, their contributions differ from those that declaratives make. The previous section showed that this has more encompassing consequences than generally acknowledged, in that all contexts should be thought of structured by including a QUD, which then is highly relevant to determine the addition to the Stalnakerian context of commonly accepted propositions by ordinary utterances of declarative sentences. Contexts thus include the Stalnakerian set of propositions to which speakers are committed in the way they are committed to their beliefs, updated by accepted assertions; but they include also a separate class of propositions to which speakers are committed in the way they are to the questions that direct their inquiry (in whatever way this is formally represented), updated by new questions and by the assertions that partially answer them. Both components are mutually known, in felicitous cases. Now, as Lewis (1969) suggested, questions can be taken as a particular kind of directive (what utterances of imperative sentences signify by default); and directives in general independently help to establish the general point we are making here. I will also use the discussion of this second, less controversial case, to confront the “flattening” strategy which opponents of the main claim I will be making tend to use to sustain their view.

Let us say first a few things about how directives should be understood in the normative framework I sketched in the second section. Alston (2000, pp. 97–103) characterizes the constitutive norm for strong directives such as orders or commands as an obligation on the addressee to carry them out, emanating from a relevant authority on the side of the speaker. Kissine (2013, ch. 4) provides a related account of directives as supplying the hearer with a (mutually manifest) reason to act. In the Williamsonian format of (KR), the constitutive condition for the specific case of ordering that these authors advance could be put like this:

(D) One must [(order A to *p*) only if one lays down on A as a result an obligation to *p*].

As in the case of the assertion norms, the obligations here in question are *sui generis* and *prima facie*. As in that case too, the combinations that the rules forbid (there, to assert what is not the case, or not known, etc.) should be possible: it should be possible to command *p* to A without A's acquiring thereby the relevant *sui generis prima facie* obligation to *p*. This requirement is met: even in the army there are specified situations under which certain orders (to perform unconstitutional acts, to violate human rights, etc.), although they come into existence as emanating from the requisite authority, are nonetheless incorrect in that the addressees do not thereby incur the intended *prima facie* obligation.

Several authors have advanced semantic accounts of *directives* on which these are semantically distinctive objects, distinct from assertions (what declarative sentences signify by default), just as questions (what interrogative sentences signify by default) are; Han (2011), Portner (forthcoming), and Jary and Kissine (2014) provide good overviews. Along the lines of Stalnaker (1978), researchers such as Han, Portner and Jary and Kissine suggest that strong directives also have a content to be added (when successful) to a collection of propositions. However, these are not those constituting the Stalnakerian common ground, but rather a “To Do List” or “Plan Set” representing something like the active projects of the addressee. This is consistent with (D); in fact, it is nicely explained by it.

Like their declarative counterparts, imperative sentences have uses that go beyond the core cases of strong directives. Uttering “take bus 44” in reply to “how do I get from here to the airport?” is not a command, but a suggestion, a piece of advice or proposal; similarly for an utterance of “come round to my house to watch the game!,” after the addressee has manifested interest in watching the game tonight and a lack of any plans for seeing it. “Help me!” is not a command, but a request. “Come in!” uttered after someone knocks on my door issues an authorization. “Get well soon!” said to someone who is ill or “Please don't rain!” looking at the sky are expressions of wishes, rather than orders. Semanticists adopt different views in light of this. Han focuses on commands as core cases, and leaves the other cases to be explained pragmatically as indirect speech acts. Portner and Jary and Kissine aim instead to provide an account general enough to encompass at least some other uses.

For our purposes, we do not need to go into these debates. We have said enough to indicate how directives add to the cumulative point we are making. Contexts are structured in complex ways, including different classes of propositions to which speakers are committed in different modes: in the way we are committed to our beliefs, but also in the way we are committed to our intentions, and to the questions guiding our inquiries. And, as we pointed out above, in felicitous contexts it is all these different commitments that are matters of mutual knowledge. As Stalnaker's (1978) account of assertion emphasizes, an accepted assertion comes to be presupposed afterwards, allowing for the satisfaction of presuppositional requirements later on in the discourse. Similarly, an accepted directive is taken for granted afterwards, constraining the legitimate moves that can be made in the discourse game, and obviously the same applies to the QUD.

Davidson (1979) and Lewis (1970) suggest dealing with non-declaratives by taking them to be synonymous with explicit performatives, and then taking the latter to have, from a semantic standpoint, the truth-conditions they appear to do compositionally. Thus, “take bus 44!” would just mean, from a semantic point of view, the proposition *that the speaker thereby requests the audience to take bus 44*. Cannot we just adopt this line and avoid having to ascribe to contexts the complex structure we have so far posited? By taking questions and directives to express the propositions self-ascribing speech-acts that these views envisage, we could just stick to the Stalnakerian view of context as a set of propositions. It will be convenient to have a label for this strategy, for we will encounter other versions of it later in our discussion. Let me refer to it as the *flattening scheme*, or simply *flattening*.

In previous work (García-Carpintero, 2004) I have argued that these views are unmotivated^10^. However, even if we accept them (perhaps invoking the sort of methodological rhetoric discussed at the end of the first section, which is not far away from Lewis's, 1970 own motivation), it is important to appreciate that such flattening will not ultimately prevent the need for extra complexity that I am advocating. Let me argue for this here, before we move to the next example where the same point may not be equally clear.

In the first place, flattening is unmotivated because the distinction between the three moods, declarative, interrogative and imperative, appears to be as semantically relevant as any syntactic distinctions can be. It is even productive and systematically reflected in English and other languages in corresponding distinctions in ascriptions of the types of acts they indicate: “I told Peter that it is raining,” “I asked Peter whether it is raining,” “I told Peter to stop the rain.” But let us grant that at a certain “core semantic” level we might disregard this, moving the distinctions to pragmatics. I have already mentioned above the debates between minimalists, moderate and radical contextualists, involving the proper account of examples such as “I am ready,” “I am tall,” “I have had breakfast,” or “there is milk in the fridge,” and have expressed my sympathies for the moderate camp (cf. García-Carpintero, 2006, 2013a). The point that makes the effect of flattening irrelevant is that, unless radicals are right, we should distinguish two kinds of pragmatic intervention. There are the processes producing clearly secondary, derivative meanings, such as particularized conversational implicatures and indirect speech acts. And there are the processes that contribute to determine what intuitively are the literal, directly conveyed meanings of ordinary utterances, as in the examples above—Bach's (1994) *implicitures*. Even if pragmatic processes are involved here, the data make it clear that they operate at a subsentential level, contributing together with the semantic compositional core to meanings that are productive and systematically determined.

So we should acknowledge three different levels of meanings, to account for which we need specific theoretical tools, reflecting three distinct robust kinds of fact. There is the *core semantic*, compositionally-driven level, at which the temporal contents of “I have had lunch” and “I have had measles” do not differ. Then there is the *secondary pragmatic* level, at which an utterance of the former sentence conveys a rejection of an invitation to go to a restaurant. And then there is the *intermediary* level of the intuitive literal and direct meaning, at which the temporal contents of the claims made by the two sentences differ—the former indicating a shorter interval between the activity and the current time. Even if pragmatic processes are involved at this intermediary level, we still need an account of it, and one that adequately interacts with the core semantic level.

As I understand views like the ones of Lewis and Davidson I am discussing, they contend that “semantics proper” (the theoretical pursuit dealing with the first level) should not care about the distinction between declaratives, interrogatives and imperatives, by invoking the flattening strategy. As I said, I do not believe this is correct. The taxonomical proposals in the debates about the semantics/pragmatics distinction I have sketched are not merely terminological arbitrary options: they have theoretical consequences. In particular, they should allow us to explain how the meanings at the intermediary level are determined, productively and systematically so, given the alleged outputs of the semantic core. It is clear that at the intermediary level a sentence in the interrogative does not mean the assertion that the speaker is asking for the relevant content, and the corresponding point applies to imperatives: they mean, respectively, a question and a directive. I believe that flattening would make it difficult to explain how the intuitively literal, direct meaning is conveyed^11^. But never mind. The important point is that the intermediary level—whether purely semantic or pragmatically intruded—is real;^12^ it systematically interacts with the core compositional determination of meaning, and we are entitled to theorize about it. Once inside it, there is no way of avoiding the complex structured contexts we have shown the need to envisage.

## Example 3: pejoratives and slurs

As announced, this is the core section of the paper; here I use the case of slurs and pejoratives to defend my main claim about the nature of contexts. Kaplan (ms^13^) started a fruitful debate on the meaning of pejoratives—as in “that bastard Kresge is famous”—including slurs and racial epithets as in “there are too many chinks in our neighborhood.” Kaplan suggests that a different dimension of expressive meaning (“use-conditional,” as opposed to truth-conditional) is required. Hom (2008) makes a case for a straightforward truth-conditional account; thus, for instance, according to him “chink” makes a truth-conditional contribution akin to that of other predicates such as “Chinese”—a property determining according to him a necessarily empty extension, which can be roughly expressed as: *ought to be subject to higher college admissions standards, and ought to be subject to exclusion from advancement to managerial positions, and …, because of being slanty-eyed, and devious, and good-at-laundering, and …, all because of being Chinese* (Hom, 2008, p. 431). As many have pointed out (cf. Jeshion, 2013a, pp. 316–319), a main difficulty for this view lies in the *projection behavior* of these terms: when sentences such as those mentioned above are negated, are antecedents of conditionals, or embedded under modal operators or in interrogative or directive mood, they still derogate the relevant targets.

To account for this, writers have argued that the expressive meaning of pejoratives and slurs is instead either a conventional implicature (Potts, 2007) or a presupposition (Macià, 2002, 2014; Schlenker, 2007)^14^. In defense of his truth-conditional account, Hom (2012, pp. 398–401) appeals to generalized conversational implicatures to explain the projection data. Now, I think a presuppositional account is more adequate; however, in order to deflate a very serious objection that has been raised against it, it is essential that we understand it relative to an extension of the proposal on the complexity of contents that I am making here. In any case, the other two proposals, the conventional implicature account and even perhaps Hom's generalized conversational implicature view, would also need to assume the extra complexity in contexts I will show we need. This is what I'll try to show in what remains of this section.

Both conventional implicatures (*that somehow being poor contrasts with being honest*, for “but” in “he is poor but honest”; *that John is married*, for the non-restrictive wh-clause in “John, who is married, will come to the party”) and presuppositions (*that someone broke the computer*, for the cleft-construction in “it was John who broke the computer”) are semantic, in that they are conventionally associated with some lexical items or constructions, and grasping them is required for full competent understanding^15^. Both are ways of conventionally indicating “non-at-issue” content. This is the most general reason why they project: thus, for instance, the negation in both “he is not poor but honest” and “it was not John who broke the computer” negates the “at issue” content, and so the same conventional implicature and presupposition as before are expressed. Neither can therefore be rejected by straightforward denials, so speakers must resort to oblique means such as Saddock's “hey, wait a minute” objection (Potts, 2012, pp. 2521–2522; Camp, 2013, pp. 341–342). Thus, it is not easy to tell them apart. Some researchers appeal to subtle projection differences (Potts, 2005; Tonhauser et al., 2013), but there is no agreement on this among linguists. In particular, their behavior when they occur in ascriptions of beliefs or acts of saying does not clearly distinguish between them, because, on the one hand, conventional implicatures might not project in such cases (Bach, 1999, pp. 338–343)^16^, as presuppositions typically do; and, on the other, presuppositions also project in some such cases, like conventional implicatures (Schlenker, 2007, p. 244)^17^.

Presuppositions and conventional implicatures have different natures (Potts, 2007, 2012). Conventional implicatures have the job of providing new information, exactly like assertions, except that it is information which (even if relevant) has a somehow background character. Felicitous presuppositions articulate (for some relevant purpose) part of what is already commonly known. Unfortunately, this again does not offer a straightforward distinction, because, as we already pointed out above with the “my wife” example, the fact that a sentence carries a presupposition can be exploited by speakers to provide uncontroversial background information, through accommodation. Nonetheless, I am convinced by the arguments by Macià and Schlenker that the data of projection and rejection, given clear-headed assumptions about the respective nature of the two phenomena, show that the best way of classifying the expressive meanings of pejoratives and slurs counts them as presuppositions.

However, probably guided by the simple-minded assumptions about context that I am questioning here, both Macià and Schlenker give an inadequate characterization of the expressive presuppositions of pejoratives, which opens the view to spurious criticism. Schlenker (2007, p. 238) offers this characterization for the slur “honky”: *the agent of the context believes in the world of the context that white people are despicable*. This is a clear-cut condition on a Stalnakerian context. But, as Williamson points out (2009, pp. 151–152), it cannot be right, because it does not capture the normative status of slurs. Exposed to utterances in the above examples, we would challenge the speaker (using perhaps some variation of the “hey, wait a minute” strategy) to retract the derogation of Kresge or Chinese people; but we would hardly challenge her to retract the suggestion that she believes that Kresge or Chinese people are despicable: for all we care, she might well believe it, but this is not what we need to dissociate ourselves from when our interlocutors utter slurs we find objectionable. As Camp (2013, p. 333) points out, Potts's conventional implicature account has the same problem, for he just posits a condition on the subjective emotional state of the speaker—something to the effect that s/he actually is in a heightened emotional state (Potts, 2007, p. 171; 2012, p. 2532)^18^.

How, then, should contexts be understood as properly capturing expressive meanings? This depends on what emotions, and the speech acts conveying them, are. What pejoratives and slurs express, in my view, is that a certain emotional state (which, as researchers on these issues have made clear, can contextually vary along different parameters, cf. Potts, 2007; Hom, 2008; Camp, 2013, among others) is *fitting* or *appropriate*. Some philosophers have argued that emotions are just a particular kind of judgment—one to the effect that an object or situation instantiates their “formal objects,” say, that Kresge or Chinese people are worthy of contempt, in our examples (cf. de Sousa, 2014; Todd, 2014, and references there). If this is right, then we do not need to go beyond the Stalnakerian context, on the assumption (which I am making) that presuppositions are ancillary speech acts, with normative essences like others, whose specific norm individuates them as requiring their contents to be common knowledge. That a speaker of “there are too many chinks in our neighborhood” takes it to be common knowledge that Chinese people are worthy of contempt explains the appropriate reaction to the utterance by non-prejudiced participants in the same conversation. Williamson (2009) seems to assume something like this^19^.

This would be a way of dealing with pejoratives analogous to the one offered by the flattening strategy for non-declaratives. Like that suggestion, however, the view of emotions and their expression on which it relies is controversial, and is rejected by many researchers (D'Arms and Jacobson, 2000, p.67; Deonna and Teroni, 2014, pp. 18–21). If emotions are instead, as I believe, *sui generis* normative states (Mulligan, 1998; D'Arms and Jacobson, 2000; Deonna and Teroni, 2014), and their expressions speech acts defined by distinctive norms, then in order to properly incorporate the presuppositional view we should add further illocutionary structure to the context set, and thus encompass them. This additional structure will be constituted by the intentional objects of the emotional states (say, Chinese people, with their (alleged) condition of generically having such-and-such features in the case of “chink”), subject to the normative condition that such intentional targets are thereby worthy of contempt and hence adequate recipients of mistreatment. On this view, the “formal object” of the emotion—the property of being contemptible in this case–is not part of the represented content, but the normative condition that allegedly justifies addressing the emotional attitude toward it.

On the suggested view of emotions and the speech acts expressing them, the additional “emotive” structure of contexts should be assumed not only on a presuppositional account of pejoratives, but also on one on which they are conventional implicatures. For, even if the expressive content of pejoratives is background but novel “information,” if unchallenged it would become part of the context set, licensing presuppositions down the line. The fact that we need to dissociate ourselves from such a prospect explains our normative reaction to utterances including slurs we disapprove of. This is why, even if Potts (2007, 2012) is right that such contents are conventional implicatures, as I mentioned above his subjective characterization of the expressive implicatures should be revised to support the present view of contexts.

Presuppositions are “filtered” in some contexts: they do not project when their triggers occur in the consequent of a conditional whose antecedent states them, or in the second conjunct of a conjunction whose first conjunct states them: *if someone broke the computer, it was John who broke it*; *someone broke the computer, and it was John who did it*. Schroeder (2014, p. 176) uses this point to dismiss the view that the expressive contents we are considering are presuppositions: “I cannot see how to construct a sentence of the form “if P, then Mark is a cheesehead” that does not implicate the speaker in disdain for people from Wisconsin.” This is well taken, but I take it to be only a consequence of the fact that the expressive contents we are discussing—be they presuppositions, or conventional implicatures—are not just forceless propositions, which is what antecedents of conditionals or conjuncts must be. This leaves open whether they are presented as requirements on the common ground (and hence have a presuppositional character), or as new background commitments (and hence are conventional implicatures). Schroeder's argument is one more example of the misleading consequences of ignoring the main claim about the nature of contexts I am making here^20^.

Some of Hom's (2010, pp. 176–179; 2012, pp. 390–391) criticisms of the presuppositional and conventional implicature view have already been discussed, or have received adequate replies in the literature. The data about projection and “cancelation” are less clear than he assumes, and in any case can be accounted for by both proposals (cf. Macià, 2014). Intuitions about the truth-values of utterances are much less clear-cut than he and others take them to be (cf. Jeshion, 2013a, 317), and again can be accounted for by both the presuppositional and the conventional implicature proposals. Hom mentions “non-orthodox” cases that lack derogatory implications; but, again, defenders of alternative views have shown them to have enough resources to deal with them, as pragmatic effects or cases of polysemy (Jeshion, 2013b, pp. 326–330). Last but not least, what Hom (2010, p. 177) thinks is the “more fundamental problem with the presupposition account” can be adequately resisted if contexts are assumed to have the sort of illocutionary complexity I am arguing for. This is how he summarizes it:

To focus on slurring as a means of efficiently entering information into the conversational record is to miss the fundamental point of slurs, namely, that they are typically used to verbally abuse their targets, with no regard to whether the negative content actually gets accommodated within a framework of rational, cooperative behavior.

He (*ibid*.) summarizes this by approvingly quoting Richard (2008, p. 21): rather than trying to enter something into the conversational record, “someone who is using these words is insulting and being hostile to their targets.” Now, the reply that the present proposal allows should be obvious. The contrast that Hom and Richard presume between making a requirement on the conversational record (or making an attempt at smuggling it there) and insulting/being hostile to some target does not exist, when what is thereby assumed to be in the context is a represented target presented as fitting the normative condition that it is contemptible and thereby liable to receive mistreatment: for this is precisely what the insult and the hostility amount to. It should be granted that Hom's and Richard's presumption that presuppositions merely concern “information” in the conversational record is shared by most of the theorists they oppose, but it is nonetheless wrong.

Actually, it is not at all obvious how Hom's own view properly captures the insulting character of utterances including slurs. His proposal is a form of the by now familiar flattening strategy for rejecting the main point of this paper in favor of straightforward truth-conditional treatments, the Davidson-Lewis line for non-declaratives, or the view that emotions are ordinary judgments, and their expression corresponding assertions. As we said, an immediate concern this raises has to do with the “projective” behavior of all such expressions under negation, conditionalization, etc.: as we have seen, intuitively expressive contents “escape” the operators under which they are embedded in such cases, while, if the expressive content is just straightforward truth-conditional content, it should remain embedded. But in fact, the problem already affects simple positive sentences: in principle, an assertion that a command is given can occur without the command being given; and an assertion that an emotional state, or the occasion for it, obtains (that something is frightening or contemptible) can equally occur without the emotional state obtaining (without the fear or contempt occurring)^21^.

As indicated above, Hom (2012, pp. 398–401) purports to explain the generation of the expressive content (in embedded and simple constructions) as a Gricean generalized conversational implicature^22^. I have serious doubts that this proposal can work on its own terms, but this need not concern us here. I want to make a point about it related to the one I made at the end of the previous section. In some cases, generalized conversational implicatures are not projected, but rather generated “locally,” i.e., interacting with the compositional determination of contents, exactly as “implicitures”/“explicitures” are. The data suggest that, in some cases, expressive contents are thus generated locally (Schlenker, 2007, p. 244). It remains to be investigated whether these should be truly handled locally by our best theories; but, if they are, a full theoretical account of the data will need to contemplate the structurally enriched contexts we have advanced, even if metaphysically/metasemantically we classify the generation of expressive contents as a generalized conversational implicature.

## Example 4: predicates of taste

The final two examples will be more cursorily discussed, but I hope they still have the power of contributing to the cumulative point I am making. In previous work with Teresa Marques (Marques and García-Carpintero, 2014), I defended a contextualist account of predicates such as “tasty” against criticisms that such a view cannot account for disagreement. In that paper we replied to proponents of recent forms of relativism, although the contextualist view that we defend is also relativist in traditional terms.

In ordinary cases of apparent disagreement between speakers who assert, respectively, *S* and *not-S*, the impression dissolves after recognition that *S* includes context-dependent expressions and that the utterances are made in different contexts. We agree, however, that an impression of disagreement remains among subjects who assent to “this is tasty” and its negation, even when it is clear that they make these judgments relative to different standards of taste. The main claim we make to account for this is that the remaining disagreement is *practical*. It concerns a non-cognitive pro-attitude that people making this sort of claim (also in thought) have, favoring shared standards, which we take to be presupposed. We take this to be a pragmatic form of a “hybrid” view of a specific set of “thick” terms^23^.

Väyrynen (2013, ch. 3–6) defends a noncommittal pragmatic view of the evaluations associated with thick concepts based on interesting data about “objectionable” concepts, on which such implications are pragmatic but are neither conventional nor conversational implicatures nor presuppositions. Objectionable thick terms are those associated with evaluations found not acceptable (such as “lewd” or “blasphemous” for many today). Väyrynen provides interesting data showing that such evaluations project under different embeddings, and also that they can be (somehow) “canceled.” As the reader will guess, however, I am not convinced by his arguments that the evaluations do not have a presuppositional status. Although he does not specifically discuss them, one of his arguments, the “appropriateness problem,” would also affect the sort of non-cognitive presuppositions we posit for terms like “tasty.” He argues (*op. cit*., 113) that someone approving the evaluation associated with “lewd” can sensibly use it while talking to commonly known objectors to such evaluations. Something similar might obtain in a case in which two food critics disagree about the food served at a restaurant, knowing full well that they do not share standards and that neither of them is at all disposed to adopt the other's standards.

However, the same situation might obtain with any ordinary presupposition, as when someone says in anger, “if the idiot I am talking to were listening, we would have less trouble.” As Stalnaker (1978, p. 87) pointed out, presupposing (or asserting it, for that matter) something that is not accepted and will not be accommodated might not be pointless, because it might well have a further point, “as Congress may pass a law knowing it will be vetoed, a labor negotiator may make a proposal knowing it will be met by a counterproposal, or a poker player may place a bet knowing it will cause all the other players to fold^24^.”

Be this as it may, properly developed, it is essential that the view is articulated in the sort of framework I am arguing for here, because according to it what is presupposed is not a proposition, but a pro-attitude: a preference for shared practical views.

## Example 5: pretense and presuppositions

In previous work (García-Carpintero, 2013c), I have defended a specific form of a “pretense-theoretic” account (alternative to Currie's, 1990 and Walton's, 1990) of fiction-makers' utterances of sentences such as “When Gregor Samsa woke, he found himself transformed into a gigantic vermin.” by Kafka in the creation of *Metamorphosis*. I defended a speech-act account, assuming the sort of Austinian view of such acts in terms of social norms in contrast to Gricean views in terms of psychological reflexive intentions I advertised above. On my proposal, while non-fictions constitutively result from *constatives*—acts of *saying*, the genus of speech acts characterized in terms of norms requiring truth for their correctness, of which assertion is the core species—fictions constitutively result from *directives*—the genus of which commands are the core species—characterized by a norm of providing the intended audience with reasons to imagine the fiction's content.

I modeled my proposal on the normative account of directives derived from Alston's (2000) outlined above in Section Example 2: Directives. As indicated there, I take commands to be subject to a norm such that they are correct only if their audiences are thereby provided with *a reason* to see to it that their content obtains. The reason itself is to be based on different sources, depending on the specific nature of the directive: the authority of the speaker in the case of commands, or the good will or presumed interests of the audience in the case of requests, suggestions, or entreaties. My proposal was that a fiction with the content *p* is a result of an act that is correct only if it gives relevant audiences (audiences of the intended kind, with the desire to engage with such works) a reason to imagine *p*. The reasons in question have to do with whatever makes engaging with good fictions worthwhile; say, to experience the succession of emotions provoked by engagement with well-drafted, suspenseful thrillers for those of us who enjoy these things, or to emotionally engage the psychological nuances that Henry James's last novels allow us to consider in depth.

Now, consider an utterance of “When Gregor Samsa woke, he found himself transformed into a gigantic vermin.” in its assumed context. This is a declarative sentence that would be used by default to make an assertion. The assertion in this case is merely pretend, which is why we would not complain that it cannot be true or impart knowledge by its including an empty name. The speaker, the fiction-maker, is using the sentence to make a different speech act, a sort of invitation or proposal to audiences of a certain kind to imagine certain contents. However, it behaves with respect to the dynamics of discourse exactly like the corresponding assertion would have done, by legitimizing presuppositions; thus, the next sentence could have been “it was not only gigantic, it was also frightening”—a cleft construction presupposing that the insect was gigantic—and it would feel entirely felicitous (as opposed to “it was not only tiny …”).

In virtue of examples like this, the common ground is not taken to consist of propositions that are strictly speaking common *knowledge*, but merely commonly “accepted” (Stalnaker, 2002). But in the framework I am advancing, we should take such an “acceptance” in the case of fiction to be a matter of further pretense: accepted pretend assertions become *pretend presuppositions*. The presuppositions, like the initial assertions, occur under the scope of a pretense. A pretend assertion is one advanced merely for its having taken place to be imagined, so that it is not subject to ordinary norms for assertions—only to norms for invitations to imagine. A pretend presupposition is similarly one put forward merely to be imagined, hence not subject to norms for presuppositions—one that does not fail because its content is not common knowledge. Fully understanding fictional discourse involves additional pretend presuppositions to the ones created by pretend assertion: the reference-fixing presuppositions that different views associate with empty names such as “Gregor Samsa” are similarly merely pretend presuppositions. It is thus irrelevant that they cannot be true, nor therefore matters of common knowledge^25^.

Not all presuppositions that a piece of fictional discourse assumes are pretend, however. Even the most fanciful tales assume facts that truly are (taken to be) common knowledge, in order to determine their contents. Special among them are presuppositions constitutive of the meaning of the terms the tale uses; these cannot be pretend. Reference-fixing presuppositions associated with names already in use also belong in this category of non-pretend presuppositions. They interact with merely pretend presuppositions to determine the content of the fiction, in ways that have been famously explored by Lewis (1978) in his influential analysis of “truth in fiction,” by Walton (1990) for his “principles of generation” and by many others under their influence. When exposed to a fiction it is very important to disentangle those presuppositions that are merely pretend from those that truly taken to be matters of common knowledge; and there is psychological evidence that the distinction is not lost on ordinary thinkers^26^.

We have thus another compelling reason to add structure to contexts, relative to what is ordinarily assumed: it is mandatory to distinguish in them propositions to which discourse participants take themselves to be committed in the way they are to their beliefs, from those to which they are merely committed in the “to be imagined” mode.

## Summary and conclusions

In this paper I have assumed a broadly Stalnakerian view of contexts, the concrete situations relative to which linguistic exchanges take place; I have assumed, that is, that they are not just sets of parameters, but meanings shared by the speakers participating in the relevant linguistic exchange. I have argued against Stalnaker's “info-centric” view of such contexts: they cannot be just propositions, or more in general representational contents, but rather these contents together with commitments toward them in different modes by speakers. This should be clear to everybody, just on the basis of the fact that conversations involve not just assertoric utterances, but also directives and questions. With respect to this familiar fact, I have made two not so familiar points: firstly, that it might well affect all sensible conversations (because all of them might involve commitments to share discourse projects); and secondly, that the familiar “flattening” strategy that attempts to reduce non-declaratives to declaratives, at least for core semantic purposes, will not make the contention ineffectual. Then, using the case of the semantics of pejoratives, I have argued that the consequences of the familiar fact are many times ignored, at the theorist's peril: flattening will not suffice in that case either; and, even among many of those who do not ignore the fact that such constructions indicate meanings additional to “at issue” contents, undue fixation on declaratives and truth-conditional content leads us to ignore the distinctive normative features of expressive meanings, providing as a result incorrect accounts. Finally, I have mentioned two other interesting examples that would allow us to sustain the same claim: the account of evaluative disagreements, and the interpretation of fictional discourse.

## Funding

Financial support for my work was provided by the DGI, Ministerio de Economía y Competitividad, Spanish Government, research project FFI2013-47948-P and Consolider-Ingenio project CSD2009-00056; and through the award *ICREA Academia* for excellence in research, 2013, funded by the Generalitat de Catalunya. Thanks to three reviewers for this journal for their comments, which led to several improvements, and to Michael Maudsley for his grammatical revision.

### Conflict of interest statement

The author declares that the research was conducted in the absence of any commercial or financial relationships that could be construed as a potential conflict of interest. The reviewer Alessandro Capone and handling Editor Alessio Plebe declared their shared affiliation, and the handling Editor states that the process nevertheless met the standards of a fair and objective review.

## Footnotes

^1^For purposes of the present contrast, I take Lewis' (1979) model as a variant of the Stalnakerian model. I mention some relevant differences below.

^2^Huvenes and Stokke (2015) question “information-centrism,” the view that context-as-body-of-information is what is needed in semantic theories of context-dependent expressions. This is also the view I am arguing against, although I will follow a different route. They use confusion cases involving indexicals and demonstratives, arguing that something beyond bodies of information is needed for proper theorizing about them. I think a more structured view of contexts along the lines to be suggested below might handle their cases, and hence that their arguments are interestingly complementary to those given here. More specifically, I think a proper handling of their cases requires adding further structure to contexts, distinguishing presuppositions that are *semantic requirements* (Fine, 2007) from those that are just shared knowledge with different sources—cp. Huvenes and Stokke (2015), fn. 12 and surrounding text.

^3^There are other notions of context in the literature, which might be free from the problems I'll raise; cf., for instance, Capone (2013), Fetzer (2012), and Gross (2001). I take it, however, that the Kaplan-Stalnaker's stance is sufficiently influential to merit discussion.

^4^This would not come as a surprise to those who contend that propositions themselves are constitutively endowed with force-like traits (King et al., 2014; Hanks, 2015); but the considerations here will not presuppose such a highly controversial view (wrong, I think), and will be compatible with more traditional views on which propositional contents themselves lack force-like features and can be put forward in different illocutionary modes.

^5^Green's (2000, p. 468) notion of the *conversational record*, defined in terms of the illocutionary commitments of discourse participants, offers a good formal model for the sort of structure I'll be arguing for.

^6^Cf. von Fintel (2008).

^7^Fricker offers two more specific reasons. First, a secondary message will be too ambiguous for the speaker to fully commit to it. Second, the audience will have to choose to draw certain inferences and it is thus they, not the speaker, who are responsible for the inferences that they choose to draw.

^8^Schaffer (2008, pp.3–5) offers a very clear, short presentation of the idea.

^9^Once again, I refer the reader to Green (2000, pp. 467–470) for a perspicuous way of formally representing the complexity I suggest we should take contexts to have, here and below.

^10^Davidsonians would do much better to adopt the “success semantics” that Ludwig (1997) and Lepore and Ludwig (2007, ch. 12) advance; when deployed in the Stalnakerian framework we are assuming, this would mean accepting the main claims I am making.

^11^I have defended that explicit performatives such as “I hereby promise not to drink again” literally say that the speaker promises by that very act not to drink again, and only indirectly convey the promise, as a form of generalized indirect speech act (García-Carpintero, 2013b). However, the account there presupposes that the three moods semantically encode information about speech-act types.

^12^I do not mean to suggest that the intermediate level is real in the straightforward psychological sense that many contextualists commit themselves to; cf. García-Carpintero (2001, 2006). However, as a reviewer helpfully suggested, it is at least real in that speakers are rationally committed to its deliverances.

^13^Kaplan, D. (ms) “The Meaning of ‘Ouch’ and ‘Oops.”’

^14^Williamson (2009) argues for a similar view. He classifies the expressive contents he proposes as conventional implicatures, but he understands that category in a traditional way, wider than the one I assume following Potts's work (*ibid*., 151, 153). I take his view to be compatible with the presuppositional account as much as with Potts's view. All these proposals can be viewer as different ways to elaborate on Kaplan's view that pejoratives should be account for by adding a “use-conditional” layer of meaning.

^15^In the case of presuppositions, Stalnaker and other writers dispute this; García-Carpintero (2015) defends it, for constructions such as the one given here for illustration.

^16^As I have pointed out elsewhere (García-Carpintero, 2006, pp.45–47), Bach (1999) in fact does not show that conventional implicatures (or presuppositions, for that matter), as understood here following Potts, are a “myth.” He only shows that they are not part of “what is said” in his “illocutionary” sense, which is just to say that they are not part of the “at issue” content of declaratives. Rather they are, according to him, part of “what is said” in his “locutionary” sense. But this just means that they are conventional, semantic in the sense that they need to be grasped for full competent understanding. This is part of current standard views on conventional implicatures, such as Potts's. Hom (2008, pp. 424–426; 2012, pp. 391–392) appears to have been misled by Bach's suggestions in his criticisms of the conventional implicature view.

^17^Ascriptions of propositional attitudes and speech acts are notoriously context-dependent; this explains the existential quantifications. In his interesting discussion of hybrid theories of evaluative terms, modeled on the views on pejoratives I am discussing, Schroeder (2009, 2014) places a strong emphasis on a distinction between hybrid expressions whose expressive content project even in attitude ascriptions, and those that do not. But, as far as I can tell, these are not properties of expressions themselves: we can only trace tendencies here. Slurs tend to project in ascriptions, but, as the examples by Schlenker and others show, they do not always do so. Such tendencies are orthogonal to the conventional implicature/presupposition divide. Quoting Bach (1999) (a work that he, unlike Hom–see previous fn.—appraises properly, cf. *op. cit*., 287-8, fn. 19), Schroeder shows that “but” might well not project in some ascriptions; but, following Potts (2005), I am taking non-restrictive wh-clauses as paradigm cases of conventional implicatures, and they do typically project in attitude ascriptions: *John said that Peter, who will be coming soon, is welcome to the party*.

^18^Boisvert (2014) provides a hybrid account of pejoratives and evaluative terms in the framework of “success” semantics, along the lines of the modified Davidsonian proposals in Ludwig (1997) and Lepore and Ludwig (2007, ch. 12). As I said before, this is better than flattening, and also (as a result) compatible with the main claims I am making here. However, like Schlenker and Potts, Boisvert assumes a psychological expressivist, non-normative account of the non-declarative additional speech acts that his account posits, which make it in my view similarly inadequate. To illustrate: there clearly is a semantic tension between uttering “thank you for *p*!” together with “shame on you for *p*!,” but this cannot be adequately captured by an account on which the sentences merely indicate that the utterer actually feels grateful and disappointed regarding *p*; for, of course, there is no inconsistency in having such feelings regarding the same situation (cp. Boisvert, 2014, p. 34). In contrast, an account on which the sentences indicate acts subject to norms such that for them to be correct the same situation is to be both worthy of gratitude and of indignation does capture the tension.

^19^Likewise, Macià (2014) poses as the expressive presupposition of “chink” *that speakers in the context are willing to treat Chinese people with a certain kind of contempt, on account of being Chinese*. This is better than Schlenker's and Potts' subjectivist proposals, but is still objectionable along the lines that I develop in the main text.

^20^It is a particularly revealing one, because it occurs in a paper that is otherwise admirably clear about the distinction between contents and forces; Schroeder's (2014, pp. 278–280) toy formal model is as clear as Green's (2000) when it comes to the proper articulation of meanings that, like expressive contents in my view, are propositions-cum-illocutionary forces.

^21^The same can intuitively obtain in the opposite direction: the non-cognitive attitude/act (the command or the derogation) can occur, without the cognitive one (the belief/assertion that the command or the derogation takes place) taking place, because the thinker/speaker lacks the conceptual resources to describe the non-cognitive state/act. Hom deals with this apparent lack of necessity of his account by appealing to semantic externalism: semantically the equivalence obtains, even if ordinary speakers lack the resources to appreciate it.

^22^The semantic externalism to which Hom appeals in order to deal with the necessity problem (see previous fn.) puts a strain on his appeal to conversational implicature to deal with this sufficiency one, because implicatures are supposed to be derivable. It is difficult to understand how ordinary speakers intuiting the allegedly implicatured condition—in our cases, the derogation of Chinese people, which is what everybody perceives in utterances of “there are too many Chinks in our neighborhood”—can make the inferences, if they themselves lack the resources to articulate the content of Hom's truth-conditional analysis.

^23^Cf. Väyrynen (2013) and Schroeder (2014) for discussion.

^24^Väyrynen's other problem for presuppositional views, the “triggering problem” (*op. cit*., 112) does not affect our proposal—which takes the relevant presupposition to be pragmatic, not lexically triggered. I am not convinced that it shows that the evaluations he discusses, associated with thick terms, are also mere pragmatic implications; but it does raise an interesting issue concerning the triggering of presuppositions—to wit, whether there are general explanations for some families of triggers (cf. Abrusán, 2011)—which I cannot address here.

^25^Cf. Sainsbury's (2010, pp. 143–148) related discussion of “truth under a presupposition.”

^26^Although the psychological data are controversial, having provided some support to those who want to deny a substantive distinction between fact and fiction (Matravers, 2014), there is also sufficient evidence supporting the phenomenological impression that we do separate what we get from fiction and non-fiction; cf. Friend (2014, forthcoming).
